# The Impact of Clinical Variables and Dialysis Modality on Kynurenine Pathway Enzymes Expression

**DOI:** 10.3390/ijms27031313

**Published:** 2026-01-28

**Authors:** Izabela Zakrocka, Katarzyna Wicha-Komsta, Sylwia Boczkowska, Renata Kloc, Tomasz Kocki, Ewa M. Urbańska, Wojciech Załuska, Andreas Kronbichler

**Affiliations:** 1 Department of Nephrology, Medical University of Lublin, 20-090 Lublin, Poland; izabela.zakrocka@umlub.edu.pl (I.Z.);; 2 Institute of Health Sciences, The John Paul II Catholic University of Lublin, Konstantynów 1F Street, 20-708 Lublin, Poland; 3 Department of Holistic Care and Management in Nursing, Medical University of Lublin, 20-081 Lublin, Poland; 4 Department of Experimental and Clinical Pharmacology, Medical University of Lublin, 20-090 Lublin, Poland; 5 Department of Internal Medicine IV, Nephrology and Hypertension, Medical University Innsbruck, 6020 Innsbruck, Austria

**Keywords:** kynurenine, kynurenic acid, tryptophan, indoleamine-2,3-dioxygenase, kynurenine aminotransferase, kynurenine-3-monooxygenase, chronic kidney disease, hemodialysis, hemodiafiltration

## Abstract

Chronic kidney disease (CKD) and kidney failure significantly reduce patients’ quality of life and markedly increase cardiovascular risk and overall mortality. Disturbed metabolism of tryptophan (Trp) through kynurenine (KYN) pathway was implicated as an important factor in kidney damage and its complications. However, the expression of genes coding crucial enzymes of KYN pathway was not examined so far. The goal of the present study was to analyze the expression of *Ido* (indoleamine-2,3-dioxygenase), *Kat1* (kynurenine aminotransferase 1), *Kat2* (kynurenine aminotransferase 2), and *Kmo* (kynurenine-3-monooxygenase) genes in patients undergoing kidney failure with kidney replacement therapy (KFRT) treatment with either hemodiafiltration (HDF) or hemodialysis (HD) in relation to selected clinical and dialysis parameters. Our data imply that *Ido*, *Kat1*, and *Kmo* gene expression does not differ between KFRT patients with analyzed comorbidities, vascular access types, or diuresis occurrence. However, *Ido* and *Kmo* gene expression correlated with pre-dialysis concentration or reduction ratio (RR) of selected metabolites. Interestingly, patients treated with HDF manifested lower *Kmo* gene expression in comparison with patients treated by HD. Our study suggests that epigenetic factors do not exert noticeable impact on the KYN pathway enzymes expression in patients with KFRT. The advantageous effect of HDF vs. HD towards the KYN pathway genes expression has potential therapeutic implications, as it may reflect superiority of the former method in KFRT patients.

## 1. Introduction

Chronic kidney disease (CKD) and its consequences became one of the major contributors of global health burden, with significant impact on quality of life, premature morbidity, and mortality [1]. CKD occurrence is estimated to continuously rise, with more patients requiring kidney failure with kidney replacement therapy (KFRT). Beyond established risk factors, disease- and therapy-related conditions significantly increase the risk of cardiovascular and all-cause mortality in individuals with KFRT. Additionally, preservation of diuresis in KFRT patients is of special importance, with a confirmed effect on overall survival [2]. KFRT, although necessary to reduce the negative impact of kidney failure, only partially restores kidney function, with effectiveness depending on the type of modality used. Despite the fact that chronic vascular inflammation and proatherogenic environment are known to be related with severe kidney damage, hemodialysis (HD) alone may mediate cardiovascular risk through immunological and complement system activation [3]. The efficacy of uremic toxins removal is one of the major indicators of HD effectiveness [4]. Hemodiafiltration (HDF) has been shown to improve patients’ survival, in particular through increased toxins clearance by convective transport, thus lowering inflammation and oxidative stress [5].

**Figure 1:**
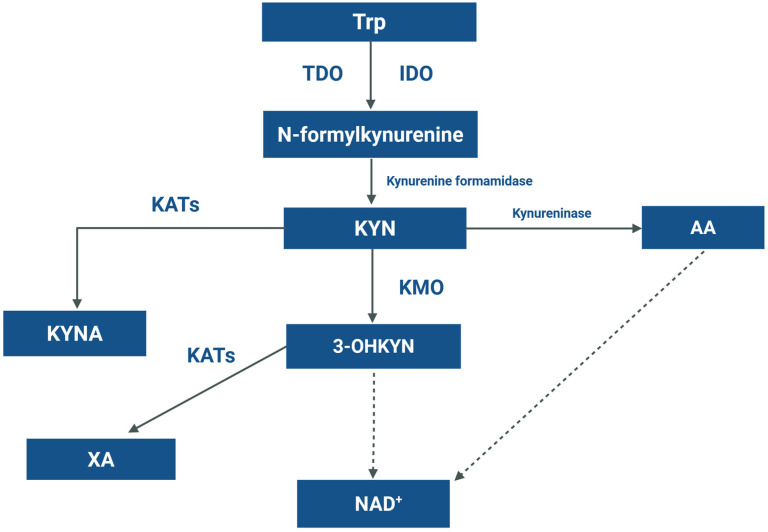
Schematic presentation of the kynurenine pathway. Abbreviations: 3-OHKYN, 3-hydroxykynurenine; AA, anthranilic acid; IDO, indoleamine-2,3-dioxygenase; KAT, kynurenine aminotransferase; KMO, kynurenine-3-monooxygenase; KYN, kynurenine; KYNA, kynurenic acid; NAD, nicotinamide adenine dinucloeotide; TDO, tryptophan 2,3-dioxygenase; Trp, tryptophan; XA, xanthurenic acid. Created in BioRender.com. Zakrocka, I. (2026) https://BioRender.com/kq81jto (accessed on 20 January 2026).

An essential aromatic amino acid tryptophan (Trp) is converted to various bioactive molecules, including serotonin, indoles, and kynurenines [6]. Beyond being a substrate for neurotransmitters and neuromodulators synthesis, over 95% of dietary Trp is catabolized through the kynurenine (KYN) pathway, yielding metabolites that impact metabolism, immune response, and cellular survival (Figure 1).

The first and rate limiting step of Trp degradation is controlled by tryptophan 2,3-dioxygenase (TDO), mainly expressed in the liver, and indoleamine 2,3-dioxygenase (IDO) found in extrahepatic tissues [7]. Conversion of Trp by TDO or IDO results in the formation of KYN, a biologically active metabolite catabolized through three different ways: (i) kynurenine aminotransferases (KATs) metabolize KYN to kynurenic acid (KYNA) [8]; (ii) secondly, KYN can be converted to anthranilic acid (AA) by kynureninase; and (iii) degradation by kynurenine-3-monooxygenase (KMO), resulting in formation of 3-hydroxykynurenine (3-OHKYN) and subsequently other active metabolites involved in the synthesis of nicotinamide adenine dinucleotide (NAD) [8].

Trp metabolites have recently been studied as potential triggers of kidney diseases [9]. Their involvement in the pathophysiology of various kidney disorders, including podocytopathies [10] or CKD [11] has been proposed. KYN metabolites level was repeatedly shown to correlate with kidney function decline, being the highest in KFRT patients [12]. Considering that multiple metabolites of KYN pathway may have an essential role in kidney function decline and its consequences, the impact of gene expression of crucial enzymes involved in Trp degradation cannot be omitted. However, an up-to-date analysis of enzymes gene expression involved in Trp metabolism was not performed in dialyzed patients.

The goal of this study was to explore the expression of genes of four major enzymes in the KYN pathway: *Ido*, *Kat1*, *Kat2*, and *Kmo* in patients dialyzed by HDF or HD. A detailed analysis of the impact of selected patients’ comorbidities, preservation of diuresis, type of vascular access, pharmacotherapy, and certain laboratory parameters on the tested genes’ expression was performed.

## 2. Results

### 2.1. Participants Characteristics

The study group consisted of 66 stable KFRT patients, with a median age of 65.5 years, and equal representation of males and females (Table 1). Glomerulonephritis was the leading cause of KFRT (38% of patients), followed by diabetes mellitus (24%), arterial hypertension (14%), and autosomal dominant polycystic kidney disease (3%). In the remaining group of patients (21%) other causes of kidney damage were reported. Among the study group the most common comorbidity was arterial hypertension (reported in 51% of patients), followed by diabetes mellitus (30%), heart failure (18%) and coronary ischemic heart disease (17%). 54% had residual kidney function (RKF). HDF was the dialysis modality performed in 38% of patients. Arteriovenous fistula was the main vascular access in the study group (67%), whereas 33% of patients were dialyzed through the central venous catheter. The median dialysis vintage was 42.5 months, with a 12 h/week dialysis treatment, and required dialysis efficacy parameters (median kT/V and urea reduction ratio of 1.52 and 73%, respectively).

### 2.2. Pre-Dialysis Level and RRs of Trp and Its Metabolites

The pre-dialysis level of Trp, KYN, KYNA, and 3-OHKYN was 8.44 µmol/L [5.92–11.37], 2.0 µmol/L [1.37–2.74], 415.15 nmol/L [307.3–593.8], and 718.15 nmol/L [477.1–966.3], respectively (Table 2).

After dialysis procedures KYN, KYNA and 3-OHKYN level significantly decreased, whereas Trp level remained unchanged (Table 2). The RR of Trp was 6.06% [−23.56–21.60}, whereas of KYN, KYNA, and 3-OHKYN it was 61.77% [43.76–74.07], 45.32% [37.95–51.33], and 68.45% [59.61–80.57], respectively.

### 2.3. Correlation Between Enzymes Gene Expression, Pre-Dialysis Metabolites Level and Their RRs

In the study group, *Ido* expression was investigated in 60 patients, *Kat1* expression in 64 patients, *Kmo* expression in 65 patients, and *Kat2* expression was only detectable in 28 patients. *Ido* expression was negatively correlated with RR of KYNA (Rho = −0.2881, *p* = 0.02). *Kat1* expression did not correlate with pre-dialysis concentration of tested KYN pathway metabolites and their RRs. *Kmo* expression negatively correlated with pre-dialysis KYNA concentration (Rho = −0.3052, *p* = 0.01), pre-dialysis 3-OHKYN concentration (Rho = −0.2850, *p* = 0.02), and RR for KYNA (Rho = −0.3843, *p* < 0.01) (Table 3).

### 2.4. Metabolites Level and Their RRs in Patients Treated with Hemodialysis and Hemodiafiltration and Their Correlation Between Enzymes Gene Expression

In patients treated with HDF, significantly lower post-dialysis KYN concentration was found compared to HD group (0.54 µmol/L vs. 0.86 µmol/L, *p* = 0.03), whereas other metabolites levels did not differ in both treated groups (Table 4).

In the subgroup analysis between HD and HDF patients, a negative correlation was found between pre-dialysis Trp concentration and *Kat1* gene expression (Rho = −0.4408, *p*= 0.03), as well as RR for KYNA and *Kmo* gene expression (Rho = −0.4392, *p* = 0.02) in HDF patients (Table 5). Additionally, in the HD subgroup a negative correlation between predialysis KYNA and *Kmo* expression was found (Rho = −0.3968, *p* = 0.01).

### 2.5. Enzymes Gene Expression in Relation to Selected Comorbidities

**Figure 2:**
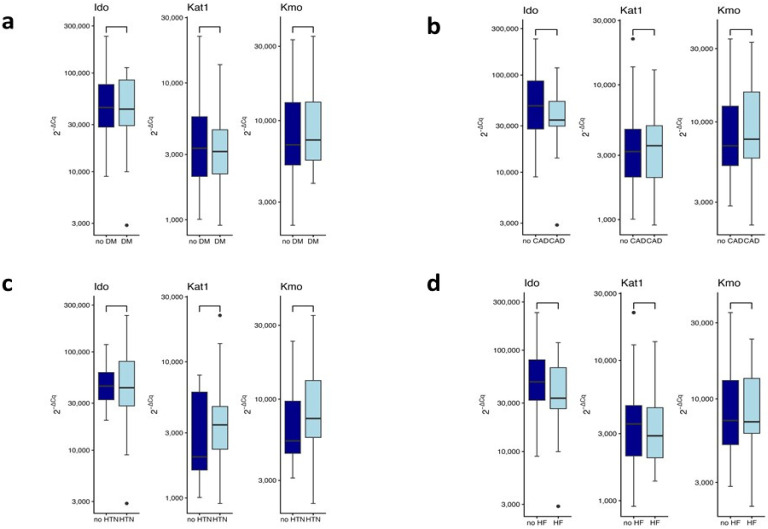
Expression of indoleamine-2,3-dioxygenase (*Ido*), kynurenine aminotransferase 1 (*Kat1*), and kynurenine monooxygenase (*Kmo*) genes in patients according to the diabetes mellitus (**a**), coronary artery disease (**b**), hypertension (**c**), and heart failure (**d**). Wilcoxon rank sum test. Data are shown as median with interquartile range. Black circles represent outliers. Abbreviations: CAD, coronary artery disease; DM, diabetes mellitus; HF, heart failure; HTN, hypertension.

There were no differences between patients with or without diabetes mellitus (DM) concerning the expression levels of *Ido* (W = 364, *p* = 0.99), *Kat1* (W = 437, *p* = 0.74), or *Kmo* (W = 381, *p* = 0.63) genes. (Figure 2a).

Additionally, no significant differences were detected in the expression of *Ido* (W = 457.5, *p* = 0.2), *Kat1* (W = 415, *p* = 0.86), or *Kmo* (W = 389, *p* = 0.47) genes when comparing patients with coronary artery disease (CAD) to individuals without this comorbidity (Figure 2b). Analysis of gene expression in the group with hypertension (HTN) vs. normotensive individuals (Figure 2c) revealed no statistically significant alterations in *Ido* (W = 237, *p* = 0.88), *Kat1* (W = 185, *p* = 0.23) or *Kmo* (W = 208, *p* = 0.26) genes expression. In patients with or without heart failure (HF) (Figure 2d), no significant differences were observed in the expression levels of *Ido* (W = 499.5, *p =* 0.17), *Kat1* (W = 478, *p* = 0.71), or *Kmo* (W = 467, *p* = 0.95) genes. Overall, these data indicate that HF does not significantly modulate the expression of the KYN pathway enzymes in the study group. These results indicate that neither DM nor CAD, HTN, or HF alter the expression of KYN pathway enzymes.

### 2.6. Enzymes Gene Expression in Relation to the Residual Kidney Function Presence

**Figure 3:**
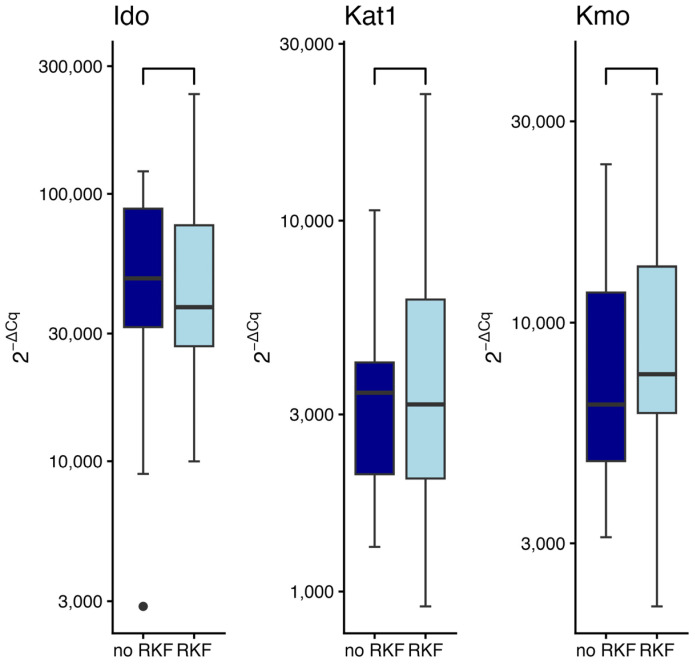
Expression of indoleamine-2,3-dioxygenase (*Ido*), kynurenine aminotransferase 1 (*Kat1*), and kynurenine monooxygenase (*Kmo*) gene in relation to residual kidney function (RKF). Wilcoxon rank sum test. Data are shown as median with interquartile range. Black circles represent outliers.

Expression of KYN pathway genes was evaluated in patients with residual kidney function (RKF) compared to those without (Figure 3). No statistically significant differences were observed for any of the examined genes: *Ido* (W = 506, *p* = 0.39), *Kat1* (W = 469, *p* = 0.61), and *Kmo* (W = 417, *p* = 0.23).

### 2.7. Enzymes Gene Expression in Patients with Different Vascular Access Type

**Figure 4:**
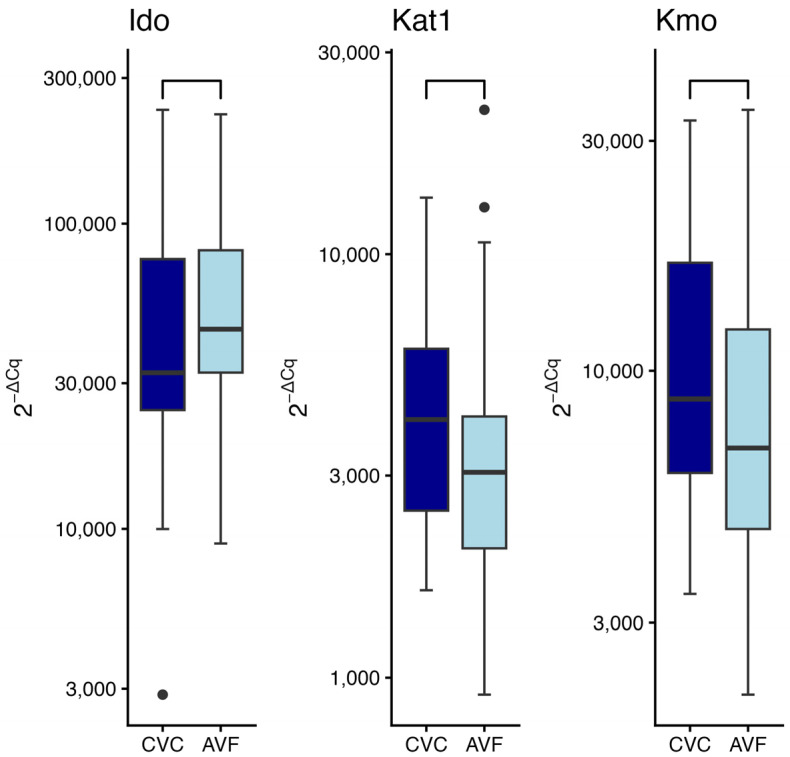
Expression of indoleamine-2,3-dioxygenase (*Ido*), kynurenine aminotransferase 1 (*Kat1*), and kynurenine monooxygenase (*Kmo*) gene in relation to vascular access type: central venous catheter (CVC) vs. arteriovenous fistula (AVF). Wilcoxon rank sum test. Data are shown as median with interquartile range. Black circles represent outliers.

The type of vascular access (arteriovenous fistula; AVF vs. central venous catheter; CVC) did not significantly influence gene expression, as determined by the Wilcoxon rank-sum test (Figure 4). No statistically significant differences were detected in the expression levels of *Ido* (W = 328, *p* = 0.26), *Kat1* (W = 552, *p* = 0.21), or *Kmo* (W = 565, *p* = 0.11) gene.

### 2.8. Enzymes Gene Expression in Relation to Dialysis Modality

**Figure 5:**
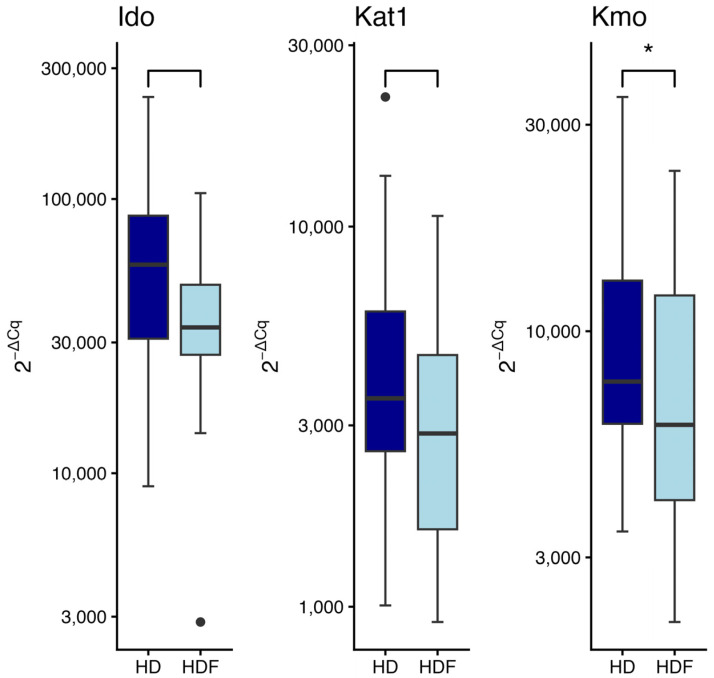
Expression of indoleamine-2,3-dioxygenase (*Ido*), kynurenine aminotransferase 1 (*Kat1*), and kynurenine monooxygenase (*Kmo*) gene in relation to different dialysis modalities: hemodialysis (HD) vs. hemodiafiltration (HDF). Wilcoxon rank sum test. Data are shown as median with interquartile range. * *p* < 0.05. Black circles represent outliers.

A significantly lower level of *Kmo* expression was observed in patients treated with the HDF technique compared to those receiving conventional HD (W = 614, *p* = 0.04) (Figure 5). A trend towards lower expression of *Ido* (W = 528, *p* = 0.06) and *Kat1* (W = 606, *p* = 0.06) gene was also found in the HDF group compared to HD patients.

## 3. Discussion

Our study revealed that the expression of all examined genes did not vary between patients with comorbidities, such as DM, CAD, HTN, or HF. The vascular access types and the RKF presence did not affect the expression of studied genes. Patients treated with HDF presented lower *Kmo* gene expression, with a tendency towards lower *Ido* and *Kat1* gene expression. *Ido* gene expression negatively correlated with RR of KYNA, while *Kmo* expression negatively correlated with pre-dialysis concentration of KYNA and 3-OHKYN, as well as with RR for KYNA. In the HD-treated group, *Kmo* gene expression negatively correlated with pre-dialysis KYNA concentration, whereas in HDF patients, *Kat1* gene expression was negatively correlated with pre-dialysis Trp level, and *Kmo* gene expression negatively correlated with RR for KYNA.

Accumulation of KYN and its metabolites in patients with severe kidney damage was postulated to be linked with endothelial dysfunction [13] and increased thrombotic risk [14,15]. It was also related with premature cardiovascular and all-cause mortality independently from traditional risk factors [16]. Additionally, serum KYN concentration was significantly associated with several concomitant diseases in CKD patients, including HF [17], coronary ischemic heart disease, or HTN [18]. In diabetic CKD patients, KYN and KYNA level correlated with kidney function decline. However, only KYN was shown to be associated with tumor necrosis factor-α concentration, but not with other pro-inflammatory markers, interleukin-6, and C-reactive protein [19]. In contrast, some studies did not confirm the association between the KYN pathway products and the risk of all-cause mortality and cardiovascular events, even after analyzing different dialysis modalities [20].

The estimations of the activity of the KYN pathway enzymes in KFRT patients were so far based on the calculated indices, such as KYN/Trp, KYNA/KYN, and 3-OHKYN/KYN ratios for IDO, KATs, and KMO activity, respectively. A direct analysis of the selected enzymes genes expression in KFRT patients was not performed up to date. Our data indicate the lack of correlation between analyzed gene expression profiles and Trp metabolites. These results imply that kidney dysfunction and uremic toxins accumulation most probably do not result from genetically determined changes of the main KYN pathway enzymes expression in KFRT patients. Impaired kidney function could be interpreted as the strongest factor related with KYN pathway products concentration in the serum compared to other diseases. Indeed, available evidence provides variable results concerning the KYN pathway enzymes in CKD animal models and in affected patients [21]. In animal models of kidney damage, an increased activity of KATs and KMO, but not of IDO, was shown in a homogenate of kidney tissue; however, enzymatic activity was not assessed in the serum [22]. In CKD patients, the activities of KATs and KMO were not studied. Serum IDO level was increased in 61 CKD patients compared to 16 healthy participants [23]. Others measured IDO protein level in peripheral blood mononuclear cells and found no correlation with KYN/Trp ratio [24].

A weak correlation of studied metabolites with the expression of genes coding for their respective biosynthetic enzymes may suggest the contribution of other factors to the formation of kynurenines. Altered toxin secretion due to impaired tubular transport and their potential post-translational impact on enzymatic activity may lead to the discrepancy between expression of an enzyme and the actual concentration of product. It is widely accepted that *Ido* expression can be induced by pro-inflammatory cytokines, resulting in a blunted immune response [25]. Interestingly, *Kmo*’s expression was shown in an animal model to increase after immunological challenge, whereas *Kat2* remained unchanged [26]. Although we found a negative correlation between *Kmo* gene expression and *Kmo*’s product level, 3-OHKYN, what may indicate a negative feedback by a 3-OHKYN on KMO’s activity, a correlation between pre-dialysis KYNA concentration and RR for KYNA, needs special attention. The direct inhibitory effect of KYNA on KMO’s activity was already shown; however, further studies should explore this association in the context of KFRT [27].

No significant differences in gene expression profile in relation to preserved diuresis were found. RKF remains a significant contributor to better quality of life, lower inflammatory state, reduced erythropoietin demand, and improved survival of KFRT patients [28]. It was recently shown that tumor necrosis factor receptor 1 (sTNFR1) level was significantly higher in dialyzed patients without preserved diuresis irrespective of adjustments for confounding parameters, again pointing towards RKF preservation as an important factor of improved outcomes in dialyzed patients [29]. However, preserved diuresis correlated negatively with pre-dialysis KYNA plasma concentration, but not with KYN, indicating that RKF remains the main way of removing selected Trp metabolites highly dependent on tubular transport [20].

In several studies, the impact of vascular access type on patients’ survival has been highlighted, with beneficial effects of AVFs compared to CVCs, mainly due to reduced risk of blood stream infections and resulting complications [30,31]. CVC use was reported to correlate with higher incidence of malnutrition–inflammation syndrome, significantly contributing to an increased risk of hospitalization and mortality [32]. Since differences between the AVF and CVC use in KFRT were reported, we hypothesized that various vascular access types may alter the KYN pathway gene expression. However, no significant differences were found, indicating a comparable rate of Trp degradation in these two groups of patients.

An ongoing debate about the advantage of HDF over HD in terms of patient outcomes remains to be answered [33]. Enhanced clearance of middle and large molecular weight solutes, with secondary reduction of inflammation and oxidative stress level translates into better quality of life, improved cognitive performance and better survival of patients remaining on HDF [34]. Here, we show that patients treated with HDF manifest lower *Kmo* gene expression compared to patients on HD. Additionally, we observed a tendency towards lower *Ido* and *Kat1* gene expression in the HDF group. This suggests lower KYN pathway activation in HDF patients, possibly having a beneficial impact on their cardiovascular and all-cause outcomes. Although no significant correlation was found between pre-dialysis KYN, KYNA, and 3-OHKYN concentration and examined genes expression in HDF treated patients, a significant negative correlation between RR for KYNA and *Kmo* gene expression in this group may suggest long term implications on this part of KYN pathway, which needs further clarifications on a larger group of patients.

There are certain limitations of our study. The experimental group included selected, stable patients dialyzed in a single facility; therefore larger multi-center studies are needed. The expression of selected genes was analyzed in patients’ lymphocytes, but not directly in the kidney tissue; therefore, we cannot exclude differences in the local gene expression of KYN pathway enzymes. Furthermore, the expression of *Kat2* was detectable only in 28 out of 66 studied patients and was therefore excluded from further analyses. Although patients were informed about general dietary recommendations, they were not on a standardized and unified diet, and the Trp-rich food intake cannot be excluded. Trp degradation by gut microbiota, resulting in KYN pathway metabolites production, could also vary between patients, although no gastrointestinal complaints were reported throughout the study. Intermittent treatment with HD or HDF results in higher variability of Trp metabolites concentration, depending on time of blood collection and not fully presenting their level in the interdialytic period. However, analytical measurements in the mid-week dialysis remain the standard procedure, making our data comparable with previous studies.

## 4. Materials and Methods

### 4.1. Study Group

The study group consisted of 66 stable patients with KFRT, receiving three times a week a bicarbonate HD (41 patients) or on-line post-dilutional HDF (25 patients). Exclusion criteria were: age < 18 years, active neoplastic process, active inflammatory conditions, any kind of hospitalization within last month, blood transfusion performed within last month, taking drugs that may affect Trp metabolism, pregnancy, breast feeding, severe comorbidity with predicted short life expectancy, single needle dialysis, dialysis through the non-tunneled catheter, or any kind of medical condition that may possibly affect signing informed written consent or taking part in the study. All HD procedures were performed on Fresenius 4008 monitors (Fresenius Medical Care, Bad Homburg, Germany), whereas HDF were conducted with post-dilution substitution mode on Fresenius 5008 monitors (Fresenius Medical Care, Bad Homburg, Germany). Ultrafiltration (UF) rate was set individually in every patient according to his interdialytic weight gain. All administered medications were thoroughly recorded and continued permanently throughout the study. Patients’ demographic and clinical data, together with the dialysis parameters (session length, total UF) were analyzed. All standard laboratory parameters were obtained before dialysis. Single pool Kt/V was established through the Daugirdas equation [35]. All participants were reminded to keep up with the recommended dietary guidelines for HD patients.

### 4.2. Sample Collection

All patients’ blood samples were obtained at the mid-week dialysis session. The level of Trp and its metabolites was assessed in blood samples collected from the arterial blood line before and after the end of each dialysis, after reducing the blood flow to 50 mL/min for 30 s and turning off the dialysate flow. Blood for genetic analyses was collected only before the dialysis procedures, since no short-term effects of the treatment performed was expected. Afterwards, blood samples for KYN pathway metabolites analysis were centrifuged for 10 min (2700 rpm), the separated plasma samples were acidified with 10% trichloroacetic acid (500 μL for each 500 μL of plasma) and again centrifuged for 10 min (12,000 rpm). Collected supernatants were kept at −72 °C for further analysis.

### 4.3. Expression of Ido, Kat1, Kat2 and Kmo Genes Analysis

Peripheral blood lymphocytes necessary for KYN pathway enzymes genes expression analysis were isolated by collecting fresh human blood into heparinized tubes, diluting it 1:1 with phosphate-buffered saline (PBS), and gently layering the mixture over Gradisol L (Aqua-Med, Łódź, Poland). The samples were then centrifuged at 2000 rpm for 20 min.

Total cellular ribonucleic acid (RNA) was isolated from lymphocytes by using RNeasy Mini QIAcube Kit (Qiagen, Hilden, Germany) and subsequently processed on the QiaCube automated workstation with an RNA isolation protocol. After RNA isolation, the concentration and purity of the cellular RNA were assessed using a Nanodrop spectrophotometer (Thermo Fisher Scientific, Waltham, MA, USA).

To obtain complementary DNA (cDNA) suitable for amplification, the RNA template was transcribed by reverse transcription. cDNA synthesis was carried out in a Veriti thermocycler using the High-Capacity cDNA Reverse Transcription Kit (ThermoFisher Scientific, Waltham, MA, USA). Into each sample containing 1 µg of RNA in 10 µL of water, 10 µL of the kit mixture was added, consisting of: 2 µL of 10× RT Buffer, 0.8 µL of 25× dNTP (deoxynucleotide triphosphates) Mix (100 mM), 2 µL of 10× RT Random Primer, 1 µL of ribonuclease inhibitor (20 U/µL), 1 µL of reverse transcriptase (50 U/µL), and 3.2 µL of ultrapure water. The samples were mixed, centrifuged, and placed in the thermocycler. The reverse transcription program consisted of the following steps: 10 min at 25 °C; 120 min at 37 °C; 5 min at 85 °C; followed by holding at 4 °C. For real-time reverse transcription polymerase chain reaction (rtPCR) gene expression analysis, a 15 µL reaction mixture was prepared, containing 1 µL of cDNA, 1.25 µL of sample, and 12.75 µL of Master Mix (ThermoFisher Scientific, Waltham, MA, USA). The following probes were used for amplification: *Kat1* (*Ccbl1*, Hs00187858_m1, FAM-MGB), *Kat2* (*Aadat*, Hs00212039_m1, FAM-MGB), *Ido* (Hs00984148_m1, FAM-MGB), *Kmo* (Hs00175738_m1, FAM-MGB), and the housekeeping gene *ACTB* encoding β-actin (Hs01060665_g1, FAM-MGB). The housekeeping gene, characterized by stable expression in tissues, was used as an internal control of the amplification process.

rtPCR reactions were performed in a 96-well plate using the QuantStudio™ 12K Flex system (ThermoFisher Scientific, Waltham, MA, USA), with a total of 40 amplification cycles. The data generated from the rtPCR reactions were analyzed using Expression Suite Software v.1.2.2 (ThermoFisher Scientific, Waltham, MA, USA). Gene expression value (RQ) of *Ido*, *Kat1*, *Kat2*, and *Kmo*, relative to *ACTB* expression value, was calculated by the formula: 2^−ΔCq^, as shown previously [36]. Since *Kat2* expression was detectable in only 28 patients, it was not taken under consideration in further analyses.

### 4.4. Serum Trp, KYN and KYNA Quantification

Patients plasma Trp, KYN, and KYNA concentrations were established through the ultra-high pressure liquid chromatography (UHPLC) (Waters Acquity UHPLC system; Waters C18 analytical column, Milford, MA, USA), as shown before [37]. Shortly, the mobile phase with 20 mM sodium acetate, 3 mM zinc acetate, and 7% acetonitrile was run with the flow rate of 0.1 mL/min. Examinations were conducted using a ultraviolet variable wavelength detector (at 250 nm for Trp and at 365 nm for KYN), and a fluorescence detector (KYNA: 344 nm excitation and 398 nm emission). The HPLC column (HR-80; 3 µm; C18 reverse-phase column) was perfused at 0.6 mL/min, with the use of mobile phase containing 2% acetonitrile, 0.9% triethylamine, 0.59% phosphoric acid, 0.27 mM sodium ethylenediaminetetraacetic acid (EDTA) and 8.9 mM heptane sulfonic acid. Standard calibration curve was prepared with the use of external standards (0.2, 0.4, 0.6, 0.8 and 1 pmol of KYNA, respectively) and showed linearity of r2 > 0.999. L-Trp (catalogue number T8941), L-KYN (sulfate salt) (catalogue number K3750), and KYNA (catalogue number K3375) were purchased from Sigma Aldrich (St. Louis, MO, USA). All necessary HPLC reagents were obtained from J.T.Baker (Avantor, Center Valley, PA, USA). Empower 3 software (Waters corporation, Milford, MA, USA, version 7.21.00.00) was used to collect and analyze HPLC data.

### 4.5. Serum 3-OHKYN Quantification

Patients’ serum 3-OHKYN level was quantified with the use of the electrochemical detector (The Thermo Scientific Dionex UltiMate 3000 ECD-3000RS, ThermoFisher Scientific, Waltham, MA, USA), connected to an analytical cell with the oxidation voltage set at 0.6 V, as shown by Heyes and Quearry [38]. Waters Spherisorb S3 ODS2 150 × 2.1 mm column (Milford, MA, USA) was perfused with a mobile phase containing 2% acetonitrile, 0.9% triethylamine, 0.59% phosphoric acid, 0.27 mM sodium EDTA, and 8.9 mM heptane sulfonic acid, with a 0.3 mL/min flow. All HPLC reagents for 3-OHKYN analysis were obtained from J.T. Baker (Avantor, Center Valley, PA, USA), whereas 3-OH-DL-KYN (catalogue number H1771) was purchased from Sigma-Aldrich (St. Louis, MO, USA). Chromeleon software (version 7.2.6, Thermo Fisher Scientific, Waltham, MA, USA) was used for HPLC system control and recording obtained chromatographic data.

### 4.6. Statistical Analysis

Reduction ratios (RRs) of Trp and its metabolites were assessed through the Bergström and Wehle equation [39]. Hemoconcentration post-dialysis concentration (Cpost) correction was conducted according to the formula:

$$corr\;Cpost=\frac{Cpost}{\left[1+\left(\frac{Bw\;pre\;-\;Bw\;post}{0.2\;Bw\;post}\right)\right]}$$

Abbreviations: corr Cpost: corrected post-dialysis concentration, Cpost: post-dialysis concentration, Bw pre: pre-dialysis body weight, Bw post: post-dialysis body weight.

RRs of metabolites examined in this study were calculated individually for each patient. Normality of the variables’ distribution was established with the use of Shapiro–Wilk test. Since all data presented a non-normal distribution, in further analyses nonparametric tests were performed. Continuous data were presented as median (interquartile range [IQR]), whereas categorical data as percentages. Due to non-normal distribution, non-parametric comparison by Wilcoxon rank sum test or Mann–Whitney test was performed. Spearman’s correlation test was conducted to analyze the correlation between the continuous variables. All statistical tests were performed in the GNU R environment (4.5.0) and STATISTICA 13.3 program. Genetic data visualization was carried out by the ggplot2 package. *p* < 0.05 was considered statistically significant.

## 5. Conclusions

The presented study indicates that patients on HDF presented significantly different genes expression compared to the HD group, with lower *Kmo* gene expression, and a trend towards lower *Ido* and *Kat1* gene expression. No significant impact of examined comorbidities, vascular access type, or residual kidney function on KYN pathway enzymes gene expression was detected. Modified expression of genes coding crucial enzymes in Trp metabolism may in part explain beneficial effect of HDF dialysis modality on patients’ survival. Further studies are necessary to assess the impact of our findings on the long-term outcomes of KFRT patients.

## Figures and Tables

**Table 1:** The characteristics of the 66 included patients. Abbreviations: DBP, diastolic blood pressure; IQR, interquartile range; SBP, systolic blood pressure; URR, urea reduction ratio; WBC, white blood cell.

Variable	N (%) or Median [IQR]
Age (years)	65.5 [55–73]
Males/females (N)	33/33
Primary cause of chronic kidney disease	
Glomerulonephritis	25 (38%)
Diabetes mellitus	16 (24%)
Arterial hypertension	9 (14%)
Autosomal dominant polycystic kidney disease	2 (3%)
Other	14 (21%)
Residual kidney function	36 (54%)
Comorbidities	
Diabetes mellitus	20 (30%)
Arterial hypertension	34 (51%)
Heart failure	12 (18%)
Coronary ischemic heart disease	11 (17%)
Hemodiafiltration	25 (38%)
Vascular access	
Arteriovenous fistula	44 (67%)
Central venous catheter	22 (33%)
Dialysis vintage (months)	42.5 [12–71]
Dialysis time (hours/week)	12 [10.5–12]
URR (%)	73 [67–75]
Kt/V	1.52 [1.35–1.74]
Pharmacotherapy	
Angiotensin converting enzyme inhibitor	11 (17%)
Angiotensin receptor blocker	1 (1.5%)
Loop diuretic	40 (61%)
Fibrate	2 (3%)
Statin	31 (47%)
Acetylsalicylic acid	24 (37%)
Erythropoiesis stimulating agent	56 (85%)
Pre-dialysis SBP (mmHg)	140 [130–157]
Pre-dialysis DBP (mmHg)	72.5 [68–80]
Hemoglobin (g/dL)	11.4 [10.5–12.2]
WBC count (10^3^/mm^3^)	6.05 [4.8–7.2]
Iron (µg/dL)	58.5 [46–68]
Transferrin (mg/dL)	169.5 [146–204]
Transferrin saturation (%)	25 [19–29]
B-type natriuretic peptide (pg/mL)	207 [103–564]
Uric acid (mg/dL)	5.30 [4.8–6.0]
C-reactive protein (mg/L)	5.45 [2.5–9.7]

**Table 2:** Pre-dialysis and post-dialysis concentrations of examined metabolites. Wilcoxon rank sum test. Data are shown as median with interquartile range. Statistically significant data are shown in bold. Abbreviations: 3-OHKYN, 3-hydroxykynurenine; KYN, kynurenine; KYNA, kynurenic acid; Trp, tryptophan.

Variable	Pre-Dialysis	Post-Dialysis	*p*
Trp (µmol/L)	8.44 [5.92–11.37]	8.46 [6.18–11.34]	0.27
KYN (µmol/L)	2.0 [1.37–2.74]	0.75 [0.46–1.02]	**<0.001**
KYNA (nmol/L)	415.15 [307.3–593.8]	230.04 [161.41–356.92]	**<0.001**
3-OHKYN (nmol/L)	718.15 [477.1–966.3]	220.33 [127.37–285.16]	**<0.001**

**Table 3:** Correlation between pre-dialysis concentration and RRs of Trp, KYN, KYNA, and 3-OHKYN and expression of *Ido*, *Kat1*, and *Kmo* in the study group. Spearman’s correlation test. * *p* < 0.05, ** *p* < 0.01. Statistically significant data are shown in bold. Abbreviations: 3-OHKYN, 3-hydroxykynurenine; *Ido*, indoleamine-2,3-dioxygenase; *Kat1*, kynurenine aminotransferase 1; *Kmo*, kynurenine monooxygenase; KYN, kynurenine; KYNA, kynurenic acid; RR, reduction ratio; Trp, tryptophan.

Variable	*Ido*		*Kat1*		*Kmo*	
	Rho	*p*	Rho	*p*	Rho	*p*
Pre-dialysis Trp	−0.0201	0.87	−0.1162	0.36	0.0513	0.68
Pre-dialysis KYN	0.0584	0.65	−0.0558	0.66	−0.0885	0.48
Pre-dialysis KYNA	−0.0939	0.47	−0.0989	0.43	**−0.3052**	0.01 *
Pre-dialysis 3-OHKYN	−0.0541	0.68	−0.1577	0.21	**−0.2850**	0.02 *
RR Trp	0.0045	0.97	−0.1087	0.39	−0.0412	0.74
RR KYN	0.0790	0.55	−0.0422	0.74	−0.0222	0.86
RR KYNA	**−0.2881**	0.02 *	−0.1152	0.36	**−0.3843**	<0.01 **
RR 3-OHKYN	−0.0138	0.91	0.0923	0.47	0.0491	0.69

**Table 4:** Comparison of pre-dialysis and post-dialysis concentrations of examined metabolites in hemodialysis (HD) and hemodiafiltration (HDF) treated patients. Mann–Whitney test. Data are shown as median with interquartile range. Statistically significant data are shown in bold. Abbreviations: 3-OHKYN, 3-hydroxykynurenine; HD, hemodialysis; HDF, hemodiafiltration; KYN, kynurenine; KYNA, kynurenic acid; Trp, tryptophan.

Variable	Pre-Dialysis			Post-Dialysis		
	HD	HDF	*p*	HD	HDF	*p*
Trp (µmol/L)	8.56 [6.77–11.25]	8.31 [5.92–11.47]	0.88	9.03 [6.55–11.88]	8.04 [5.75–10.10]	0.46
KYN (µmol/L)	1.91 [1.34–2.78]	2.07 [1.55–2.39]	0.67	0.86 [0.52–1.34]	0.54 [0.31–0.92]	**0.03**
KYNA (nmol/L)	396 [269.8–520.5]	488.6 [321.3–746.3]	0.14	217.2 [161.41–327.01]	236.88 [165.92–428.46]	0.37
3-OHKYN (nmol/L)	640.9 [504.9–934.2]	745.3 [477.1–968.3]	0.60	224.04 [158.83–314.22]	204.94 [127.37–246]	0.42

**Table 5:** Correlation between pre-dialysis concentration of Trp, KYN, KYNA, and 3-OHKYN, their RRs, and expression of *Ido*, *Kat1*, and *Kmo* in patients treated with hemodialysis (HD) or hemodiafiltration (HDF). Spearman’ correlation test. * *p* < 0.05. Statistically significant data are shown in bold. Abbreviations: 3-OHKYN, 3-hydroxykynurenine; HD, hemodialysis; HDF, hemodiafiltration; *Ido*, indoleamine-2,3-dioxygenase; *Kat1*, kynurenine aminotransferase 1; *Kmo*, kynurenine monooxygenase; KYN, kynurenine; KYNA, kynurenic acid; RR, reduction ratio; Trp, tryptophan.

Variable	HD						HDF					
	*Ido*		*Kat1*		*Kmo*		*Ido*		*Kat1*		*Kmo*	
	Rho	*p*	Rho	*p*	Rho	*p*	Rho	*p*	Rho	*p*	Rho	*p*
Pre-dialysis Trp	0.1725	0.30	0.0515	0.75	0.1749	0.27	−0.2242	0.31	**−0.4408**	0.03 *	−0.1582	0.46
Pre-dialysis KYN	0.1298	0.43	−0.0732	0.65	−0.1906	0.23	−0.1790	0.42	−0.0930	0.66	0.6654	0.81
Pre-dialysis KYNA	−0.1040	0.53	−0.2313	0.15	**−0.3968**	0.01 *	−0.0971	0.66	0.2200	0.30	−0.1626	0.44
Pre-dialysis 3-OHKYN	0.0966	0.56	−0.0727	0.65	−0.1158	0.47	−0.2813	0.20	−0.1900	0.37	−0.3565	0.08
RR Trp	0.1506	0.36	0.0493	0.76	0.0789	0.62	−0.0587	0.79	−0.3252	0.12	−0.0815	0.69
RR KYN	0.0669	0.70	−0.1780	0.29	−0.1195	0.47	0.1632	0.46	0.3747	0.07	0.35	0.08
RR KYNA	−0.2800	0.08	0.0823	0.61	−0.1763	0.27	−0.3880	0.07	−0.2217	0.29	**−0.4392**	0.02 *
RR 3-OHKYN	0.0263	0.87	0.1287	0.42	0.1918	0.22	0.0383	0.86	0.2509	0.24	−0.0104	0.96

## Data Availability

The original contributions presented in this study are included in the article. Further inquiries can be directed to the corresponding author.

## Abbreviations

The following abbreviations are used in this manuscript:

3-OHKYN	3-hydroxykynurenine
AA	anthranilic acid
AVF	arteriovenous fistula
Bw pre	pre-dialysis body weight
Bw post	post-dialysis body weight
CAD	coronary artery disease
cDNA	complementary DNA
CKD	chronic kidney disease
Corr Cpost	corrected post-dialysis concentration
Cpost	post-dialysis concentration
CVC	central venous catheter
DBP	diastolic blood pressure
DM	diabetes mellitus
dNTP	deoxynucleotide triphosphates
EDTA	ethylenediaminetetraacetic acid
HD	hemodialysis
HDF	hemodiafiltration
HF	heart failure
HPLC	high performance liquid chromatography
HTN	arterial hypertension
Ido	indoleamine-2,3-dioxygenase
IQR	interquartile range
KAT	kynurenine aminotransferase
Kat1	kynurenine aminotransferase 1
Kat2	kynurenine aminotransferase 2
KFRT	kidney failure with kidney replacement therapy
Kmo	kynurenine monooxygenase
KYN	kynurenine
KYNA	kynurenic acid
NAD	nicotinamide adenine dinucleotide
PBS	phosphate-buffered saline
RKF	residual kidney function
RNA	ribonucleic acid
RQ	gene expression value
RR	reduction ratio
rtPCR	reverse transcription polymerase chain reaction
SBP	systolic blood pressure
sTNFR1	soluble tumor necrosis factor receptor 1
TDO	tryptophan 2,3-dioxygenase
Trp	tryptophan
UF	ultrafiltration
UHPLC	ultra-high pressure liquid chromatography
URR	urea reduction ratio
WBC	white blood cell
XA	xanthurenic acid
